# PGC-1α integrates insulin signaling with mitochondrial physiology and behavior in a Drosophila model of Fragile X Syndrome

**DOI:** 10.1038/s44324-024-00004-7

**Published:** 2024-02-21

**Authors:** Eliana D. Weisz, Adam R. Fenton, Thomas A. Jongens

**Affiliations:** 1 Department of Genetics, Perelman School of Medicine at the University of Pennsylvania, Philadelphia, PA 19104, USA.; 2 Autism Spectrum Program of Excellence, Perelman School of Medicine at the University of Pennsylvania, Philadelphia, PA 19104, USA.

## Abstract

Fragile X Syndrome (FXS) is the most prevalent monogenetic form of intellectual disability and autism. Recently, dysregulation of insulin signaling (IS) and aberrations in mitochondrial function have emerged as robust, evolutionarily conserved components of FXS pathophysiology. However, the mechanisms by which altered IS and mitochondrial dysfunction impact behavior in the context of FXS remain elusive. Here, we show that normalization of IS improves mitochondrial volume and function in flies that lack expression of *dfmr1*, the *Drosophila* homolog of the causal gene of FXS in humans. Further, we demonstrate that dysregulation of IS underlies diminished expression of the mitochondrial master regulator PGC-1α/Spargel in *dfmr1* mutant flies. These results are behaviorally relevant, as we show that pan-neuronal augmentation of PGC-1α/Spargel improves circadian behavior in *dfmr1* mutants. Notably, we also show that modulation of PGC-1α/Spargel expression in wild-type flies phenocopies the *dfmr1* mutant circadian defect. Taken together, the results presented herein provide a mechanistic link between mitochondrial function and circadian behavior both in FXS pathogenesis as well as more broadly at the interface between metabolism and behavioral output.

Fragile X Syndrome (FXS) is the most common monogenetic cause of intellectual disability and autism^1,2^. Affected individuals experience a variety of difficulties that generally preclude their ability to care for themselves and create many challenges for patients and caretakers alike^3–8^. Currently, there is no cure for FXS. Rather, a better understanding of FXS pathogenesis is necessary to identify effective treatments that improve the capabilities and quality of life of FXS patients.

At the molecular level, FXS is caused by loss of function of the *FMR1* gene^9–12^. The protein product of *FMR1*, termed FMRP, is an RNA binding protein. While FMRP was initially defined as a translational repressor, advancements in cell-type specific transcriptomic and proteomic approaches have enabled us to learn that FMRP can also promote the expression of many of its targets through multiple modes of gene regulation^13–19^.

Since the etiology of FXS is well characterized, researchers have been able to generate valuable preclinical disease models^3,20,21^. With the advent of a wealth of genetic tools, *Drosophila melanogaster* has emerged as a highly tractable model for the study of FXS^22–26^. The *Drosophila* genome encodes a single gene, *dfmr1*, that is the sole ortholog of the FMR protein family^27^. The product of *dfmr1*, termed dFMRP, shares both sequence identity and biochemical properties with those of its mammalian ortholog^27^. Importantly, flies that harbor loss-of-function mutations in the *dfmr1* gene recapitulate many characteristics of FXS. Specifically, *dfmr1* mutant flies have neuroanatomical defects as well as deficits in memory, social behavior, and circadian rhythms^22–24,28–31^.

Studies in preclinical models of FXS have uncovered signaling molecules and pathways that are dysregulated in the disease state. Previously, we reported that several components of the insulin signaling (IS) pathway are elevated in the brains of *dfmr1* mutants^32^. These changes included increased levels of the major insulin-like peptide, elevated phosphoinositide 3-kinase (PI3K) activity, and accumulation of phosphorylated Akt at the plasma membrane^32^. Aberrant IS appears to be directly linked to behavioral and cognitive function, as genetic manipulations that reduced IS were sufficient to increase circadian rhythmicity and rescue memory deficits in our *Drosophila* FXS model^32^. Dysregulation of IS has also been identified as an evolutionarily conserved feature of FXS pathogenesis in murine and patient derived cell models^32–40^. Moreover, a multitude of genetic and pharmacological approaches that normalize insulin signaling are sufficient to restore behavior and cognition in mammalian FXS models^32–39,41^. However, the mechanism by which altered IS impinges on behavioral and cognitive function in FXS remains elusive.

To better understand the mechanism by which dysregulation of IS impacts behavioral and cognitive outputs in the *dfmr1* mutants, we conducted an unbiased metabolomics analysis. Paradoxically, our metabolic studies revealed reduced levels of carbohydrate and lipid metabolites in the *dfmr1* mutants despite increased brain IS; hyperphagia; and normal body size and activity levels^42^. We also found a robust decrease in the redox ratio of the mitochondrial cofactor nicotinate adenine dinucleotide (NAD^+^/NADH) and qualitative defects in mitochondrial ultrastructure^42^. In aggregate, these robust metabolic defects suggest that mitochondrial function is impaired in our *Drosophila* model of FXS.

The notion that mitochondrial dysfunction underlies behavioral and cognitive impairments in preclinical models of FXS is particularly compelling in light of recent studies that have implicated mitochondrial defects in the pathogenesis of intellectual disability related syndromes and autism^43–45^. While the central nervous system (CNS) represents 2% of total body weight, it consumes around 20% of inspired oxygen at rest^43^. This high oxidative demand renders the CNS particularly sensitive to changes in mitochondrial metabolism^43^. Proper mitochondrial function is especially critical for the establishment of neuronal connectivity, neurogenesis, and synaptic plasticity^43,46^.

In this study, we characterize alterations of mitochondrial morphology and function in *dfmr1* mutant flies and demonstrate that genetic reduction of IS is sufficient to alleviate these mitochondrial defects. Together, these findings suggest that restoration of mitochondrial function is a mechanism by which normalization of IS improves behavior and cognition in a preclinical model of FXS. Moreover, we present evidence that elevated IS underlies diminished expression of the mitochondrial master regulator, PGC-1α, in the heads of *dfmr1* mutants. We report that pan-neuronal augmentation of PGC-1α in *dfmr1* mutants is sufficient to restore circadian behavior. Beyond their translational relevance for the FXS field, our results indicate a novel role of PGC-1α in the regulation of circadian rhythmicity and thereby provide a mechanism by which metabolism and mitochondrial function inform behavioral output.

## Results

### Mitochondrial volume and function are diminished in the absence of dFMRP

**Fig. 1 |:**
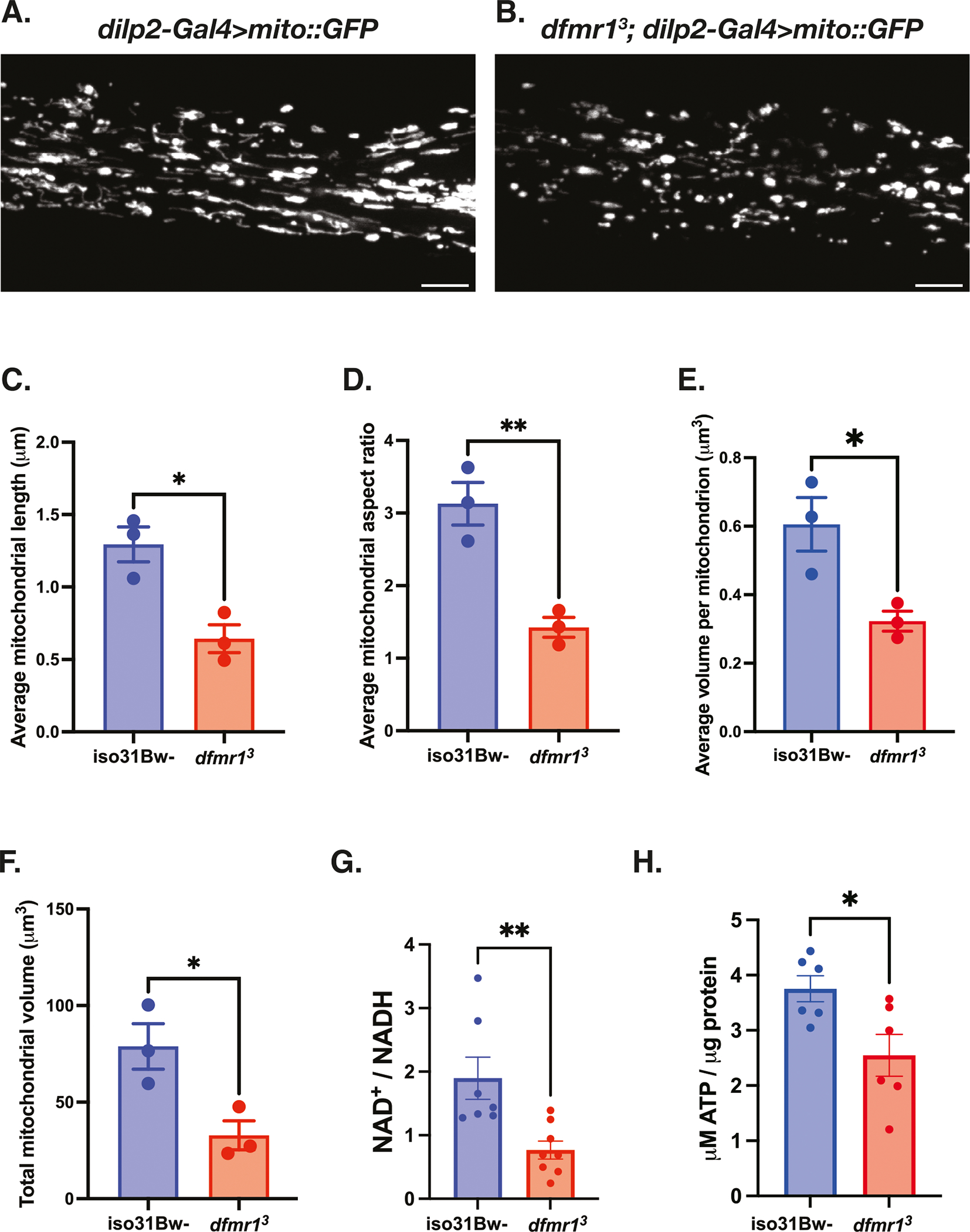
Mitochondrial volume and function are compromised in the absence of dFMRP. **A–F** Mitochondria in the insulin producing cells (IPCs) of the brain were labeled by expressing a genetically encoded *UAS*-*mitoGFP* construct in conjunction with the *dilp2-Gal4* driver. Representative maximum-intensity projections of GFP-labeled mitochondria in the IPC processes of (**A**) *iso31Bw*- wild type and (**B**) *dfmr1* mutant flies. Scale bars: 5 μm. Images are oriented with the dorsal side on the left and the ventral side on the right. Quantification of (**C**) average mitochondrial length, (**D**) average mitochondrial aspect ratio, (**E**) average volume per mitochondrion, and (**F**) average total mitochondrial volume per brain. Sample number (N) per genotype = 3 brains. Unpaired t-tests indicated that the average length, aspect ratio, volume per mitochondrion, and total mitochondrial volume per brain were all significantly reduced in *dfmr1* mutants compared to *iso31Bw*- wild type controls. Values represent mean ± SEM. *p ≤ 0.05, **p ≤ 0.01. **G** Quantification of the NAD^+^/NADH ratio. Each sample contained 10 fly bodies. Sample number (N) per genotype: *iso31Bw*- = *7*, *dfmr1* = 8. An unpaired t-test indicated that the NAD^+^/NADH ratio was significantly diminished in *dfmr1* mutants compared to *iso31Bw*- wild type controls (*p* = 0.0059). Values represent mean ± SEM. **H** Quantification of ATP levels relative to protein content. Each sample contained 5 fly bodies. Sample number (N) per genotype = 6. An unpaired t-test showed that ATP levels were significantly decreased in *dfmr1* mutants compared to iso31Bw- wild type controls (*p* = 0.0224). Values represent mean ± SEM.

Prompted by our previous discovery that mitochondria in the indirect flight muscle of *dfmr1* mutants appeared to be qualitatively smaller and irregularly shaped, we wanted to quantitatively examine mitochondrial morphology in behaviorally relevant tissues. To do so, we leveraged a genetically encoded chimeric GFP construct (*UAS-mitoGFP*) that allowed us to label mitochondria in specific subtypes of cells with the binary Gal4/UAS system^47^. We were particularly interested in visualizing mitochondria in the insulin producing cells (IPCs) of the brain, because we previously found that constitutive expression of the *UAS-dfmr1* transgene under the control of a *dilp2-Gal4* driver that is specific to the 14 IPCs of the brain was sufficient to restore normal circadian behavior in *dfmr1* mutants^32^. Therefore, we expressed the *UAS-mitoGFP* transgene in conjunction with the *dilp2-Gal4* driver and used confocal microscopy to resolve GFP-labeled mitochondria in the IPC processes that extend ventrally through the brain. We observed that mitochondria in the IPCs of *dfmr1* mutants were shorter than controls, with decreased length and aspect ratio relative to *iso31Bw*- wild-type controls (Fig. 1A–D). Further, the average volume of individual mitochondria and total mitochondrial volume was decreased in the brains of *dfmr1* mutants compared to controls (Fig. 1E, F). These findings match recent studies that revealed small mitochondria with decreased aspect ratios in the brains of *Fmr1*^*−/y*^ mice during development and synaptic maturation^48,49^.

Considering that mitochondrial network morphology and bioenergetic capacity are intimately intertwined, we next measured ATP levels in *dfmr1* mutants and wild type conspecifics as a direct physiologic readout of mitochondrial function. Consistent with the observed defects in mitochondrial morphology, we found that ATP levels were diminished in the *dfmr1* mutant flies compared to wild-type controls (Fig. 1H). Given that the ability of mitochondria to generate ATP is dependent on the redox state of the cofactor NAD^+^, the diminution of ATP levels that we observed corresponds with our previous discovery^42^, which we have confirmed herein, that the NAD^+^/NADH ratio is significantly decreased in *dfmr1* mutants (Fig. 1G). Our findings are also consistent with several reports of diminished cytosolic ATP levels in murine models of FXS^48–51^. Collectively, it appears that the mitochondria of *dfmr1* mutant flies are characterized by aberrant mitochondrial network morphology, decreased ATP levels, and a diminished NAD^+^/NADH ratio.

### Genetic reduction of IS ameliorates mitochondrial volume and function in *dfmr1* mutants

Once we established that the *dfmr1* mutants exhibit several robust hallmarks of mitochondrial dysfunction, we then sought to better understand the contribution of mitochondrial dysfunction to FXS pathophysiology. For these experiments, we introduced one copy of a null allele of the *dilp2* gene, which encodes the most abundant *Drosophila* insulin-like peptide, into the *dfmr1* mutant background. We were particularly interested in reduction of *dilp2* gene dosage because we have previously demonstrated that this precise genetic manipulation restores behavioral and cognitive function in the *dfmr1* mutants^32^. As an initial indication of mitochondrial health, we conducted TEM experiments on longitudinal sections of isolated thoraces to visualize the impact of genetic reduction of insulin signaling on mitochondrial ultrastructure in *dfmr1* mutant flies as well as wild-type controls. We selected the thorax for TEM analysis because this tissue has a distinctive structure of mitochondria aligned along myofibrils to support the high energy demand of flight^52^. Encouragingly, we observed that in contrast to the small, irregularly shaped mitochondria present in micrographs from *dfmr1* mutants, elimination of one copy of the *dilp2* gene in *dfmr1* mutants restored mitochondrial ultrastructure (Supplementary Fig. 1).

**Fig. 2 |:**
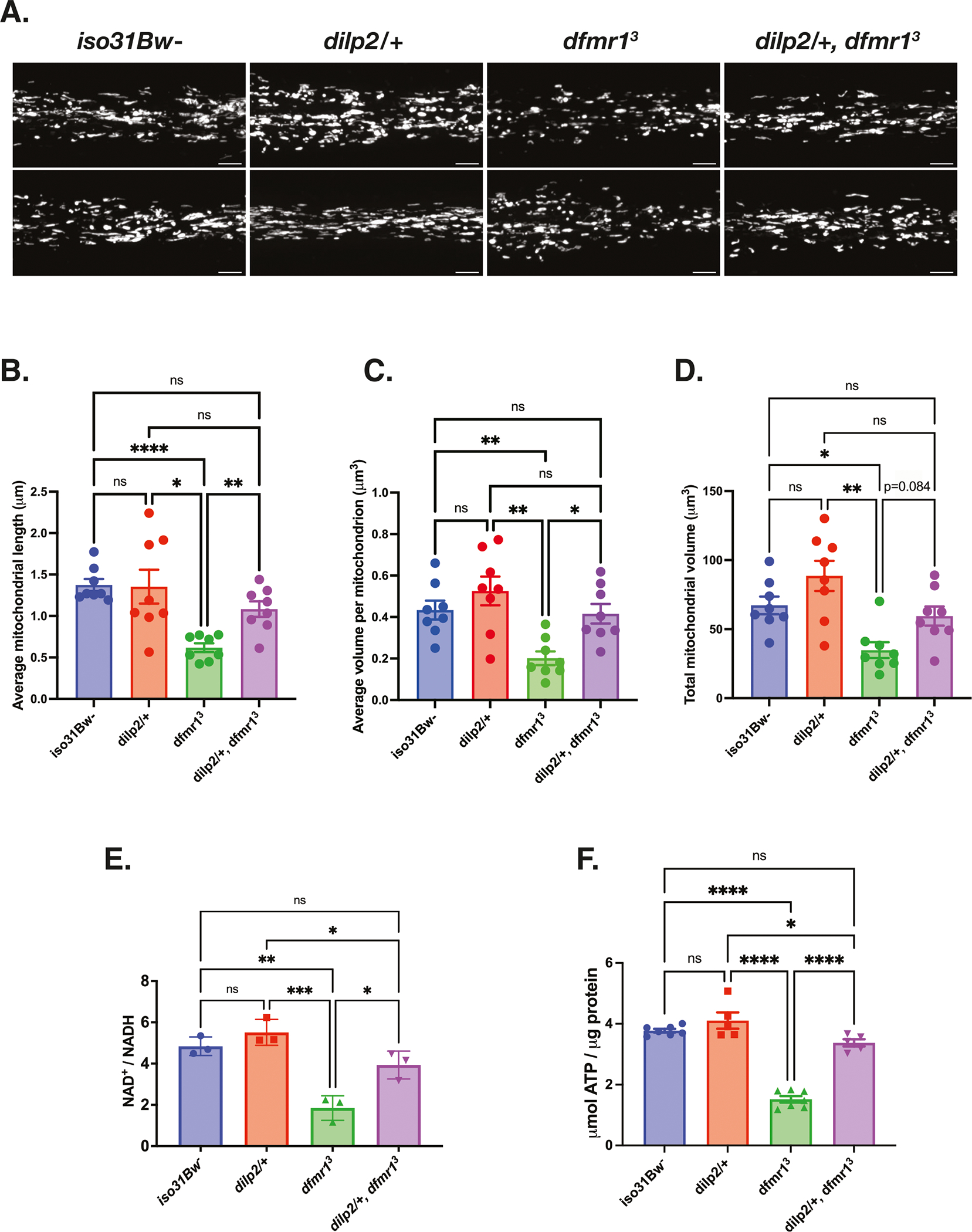
Genetic reduction of IS augments mitochondrial volume and function in *dfmr1* mutant flies. **A–D** Mitochondria in the insulin producing cells (IPCs) of the brain were labeled by expressing a genetically encoded *UAS-mitoGFP* construct in conjunction with the *dilp2-Gal4* driver. **A** Two representative maximum-intensity projections of GFP-labeled mitochondria in the IPC processes of *iso31Bw*- wild type, *dilp2/+* heterozygous mutant, *dfmr1* homozygous mutant, and *dilp2*/+, *dfmr1* double mutant flies. Scale bars: 5 μm. Images are oriented with the dorsal side on the left and the ventral side on the right. Sample number (N) per genotype = 8 brains. Brown-Forsythe and Welch ANOVA with Dunnet’s T3 multiple comparisons test revealed that genetic reduction of insulin signaling significantly improved (**B**) average mitochondrial length and (**C**) average volume per mitochondrion. **D** Total mitochondrial volume was not significantly increased in *dilp2*/+, *dfmr1* double mutants, but showed a trend towards improvement (*p* = 0.084). Values represent mean ± SEM. *p ≤ 0.05, **p ≤ 0.01, ***p ≤ 0.001, ****p < 0.0001. (**E**) Quantification of the NAD^+^/NADH ratio.^.^ Each sample contained 10 fly bodies. Sample number (N) per genotype = 3. One-way ANOVAs revealed a significant group effect for the NAD^+^/NADH ratio (*p* = 0.0003). Post hoc Tukey tests indicated that while *dfmr1* mutant flies had a significantly lower NAD^+^/NADH ratio than their *iso31Bw*- and *dilp2*/+ heterozygous mutant conspecifics, *dilp2*/+,*dfmr1* double mutant flies had a significantly improved NAD^+^/NADH ratio. Values represent mean ± SEM. *p ≤ 0.05, **p ≤ 0.01, ***p ≤ 0.001. **F** Quantification of ATP levels relative to protein content. Each sample contained 5 fly bodies. Sample number (N) per genotype: *iso31Bw*- = 6, *dilp2*/+ = 5, *dfmr1* = 6, *dilp2/*+, *dfmr1* = 5. One-way ANOVAs revealed a significant group effect for ATP levels (*p* < 0.0001). Post hoc Tukey tests indicated that while *dfmr1* mutant flies had a significantly lower ATP levels than their *iso31Bw*- and *dilp2*/+ mutant conspecifics, *dilp2*/+,*dfmr1* double mutant flies had a significant boost in ATP levels. Values represent mean ± SEM. *p ≤ 0.05, ****p ≤ 0.0001.

To quantitatively assess the impact of genetic reduction of IS on the mitochondrial network of *dfmr1* mutants, we generated *dilp2/*+, *dfmr1* double mutant flies in which mitochondria in the IPCs were labeled with GFP (*dilp2-Gal4 > UAS-mitoGFP*). Congruent with our TEM findings, we observed that the average length and volume per mitochondrion in the IPCs of *dilp2*/+, *dfmr1* double mutant flies was significantly increased relative to *dfmr1* single mutants (Fig. 2A–C). Additionally, the total mitochondrial volume of *dilp2*/+, *dfmr1* double mutants was indistinguishable from that of *iso31Bw*- control flies (Fig. 2D). Taken together, the results of our TEM and confocal microscopy studies clearly demonstrate that genetic reduction of IS ameliorates brain and peripheral mitochondrial network morphology.

Given that normalization of IS substantially improved mitochondrial ultrastructure and network morphology in the *dfmr1* mutant flies, we postulated that constitutive elimination of one allele of the *dilp2* gene would also ameliorate mitochondrial function in *dfmr1* mutants. As predicted, we found that *dfmr1* mutants that carried one null allele of the *dilp2* gene had a significantly higher whole-body NAD^+^/NADH ratio and ATP levels than their *dfmr1* mutant counterparts (Fig. 2E, F). The rescue of mitochondrial defects by a manipulation that restores circadian rhythmicity and memory in *dfmr1* mutant flies suggests that these processes are mechanistically linked. Thus, our results indicate that restoration of mitochondrial function is a potential mechanism by which normalization of IS improves behavior and cognition in the *Drosophila* model of FXS.

### Elevated IS inhibits mitochondrial function by repression of Spargel/ PGC-1α expression in *dfmr1* mutants

One intriguing candidate that integrates cellular energy metabolism with mitochondrial biomass and function is PGC-1α expression. Diminished PGC-1α expression is a compelling explanation for the bioenergetic defects in FXS because the PGC-1 family of proteins are transcriptional coactivators that strongly induce mitochondrial biogenesis and function^53^. Alignment of the *Drosophila* Spargel (Srl) protein with its three mammalian homologs revealed a high degree of sequence identity, particularly with respect to functional domains^53^. Similar to its mammalian counterpart, Srl is a transcriptional coactivator that has been shown to modulate mitochondrial biogenesis and energy metabolism^53,54^. Thus, we can leverage the *Drosophila* model system to circumvent the functional redundancy present in mammalian models and identify possible roles of PGC-1α/Srl in FXS pathogenesis. Remarkably, flies with loss-of-function mutations in *Srl* have several phenotypic commonalities with *dfmr1* mutants including, similar deficits in energy stores; fragmented mitochondria; locomotor impairment; decreased female fecundity; and shortened lifespan^25,42,54–56^. The robust phenotypic overlap between *dfmr1* and *Srl* loss-of-function suggests that these genes act in a common pathway to modulate mitochondrial function, behavior, and cognition.

**Fig. 3 |:**
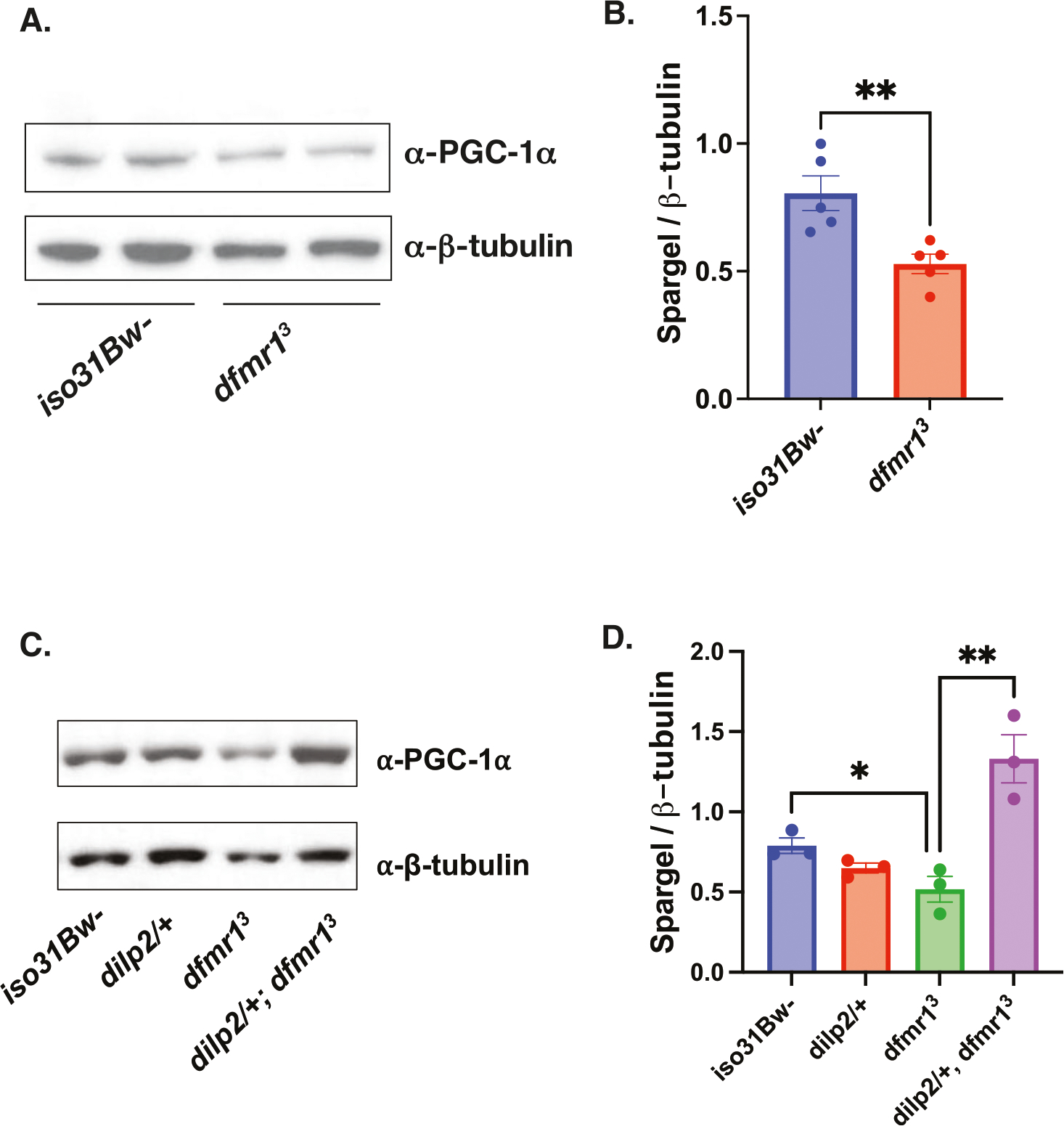
Diminished Spargel expression in the heads of *dfmr1* mutants is restored by normalization of IS. **A** Western analysis of Spargel expression in extracts from *iso31Bw*- and *dfmr1* mutant fly heads. An antibody to PGC-1α was used to detect Spargel expression (top). β-Tubulin was used as a loading control (bottom). See Supplementary Fig. 3 for full blots. **B** Quantification of the intensity of Spargel relative to β-Tubulin. An unpaired t-test revealed that Spargel levels are diminished in *dfmr1* mutant heads compared to *iso31Bw*- controls (*p* = 0.0074). Sample number (N) per genotype = 5. Each sample contained 10 fly heads. Values represent mean ± SEM. **p ≤ 0.01. **C** Western analysis of Spargel expression in extracts from *iso31Bw*- wild type, *dilp2*/+ heterozygous mutant, *dfmr1* homozygous mutant, and *dilp2*/+, *dfmr1* double mutant fly heads. See Supplemental Fig. 4 for full blot. **D** An unpaired t-test indicated that *dilp2*/+, *dfmr1* double mutant flies have higher Spargel expression than *dfmr1* single mutants (*p* = 0.0088). Sample number (N) per genotype = 3. Each sample contained 10 fly heads. Values represent mean ± SEM. **p ≤ 0.01.

To measure Srl protein levels in the heads of *dfmr1* mutant and wild-type flies, we first validated that the mouse monoclonal antibody that specifically recognizes endogenous forms of PGC-1α cross-reacts with the *Drosophila* Srl protein (Supplementary Fig. 2). As predicted by the convergence of loss-of-function phenotypes and the expected effects of known cellular signaling defects, we observed that Srl protein levels are diminished in the heads of *dfmr1* mutants compared to wild-type controls (Fig. 3A, B, Supplementary Fig. 3). This finding is congruent with a previous report that PGC-1α transcript levels are significantly down-regulated in an unbiased transcriptomic analysis of hippocampal neurons in *Fmr1*^−/y^ mice^57^.

To determine whether elevated IS contributes to decreased Srl expression, we measured Srl levels in the heads of *dfmr1* mutants that carried a null allele of *dilp2*. We observed that genetic reduction of IS restored Srl expression in the heads of *dfmr1* mutants to wild-type levels (Fig. 3C, D, Supplementary Fig. 4).As anotherindependent manipulation of IS, we acutely administered a highly specific PI3K inhibitor, LY294002, for 5 days post-eclosion and quantified Srl expression in the heads of *dfmr1* mutants and wild-type controls. Similar to our findings with genetic reduction of IS, we observed that pharmacologic reduction of IS boosts Srl expression in the heads *dfmr1* mutants (Supplementary Fig. 5).

### Pan-neuronal augmentation of Spargel expression rescues circadian rhythmicity in the *dfmr1* mutants

Next, we queried whether elevation of Srl expression is sufficient to improve behavioral defects in the *dfmr1* mutant flies. To genetically boost Srl expression in *dfmr1* mutants, we used enhancer promoter (EP) induced overexpression of the *Srl* locus in the CNS. We obtained flies that contain a UAS element upstream of the endogenous *Srl* gene, termed *Srl*^*EY05931*^, and made recombinants in the *dfmr1* mutant background. The resultant progeny were crossed to *dfmr1* mutants that expressed the *elav-Gal4* pan-neuronal driver to generate *dfmr1* mutant flies that expressed the *Srl*^*EY05931*^ transgene in conjunction with the *elav-Gal4* pan-neuronal driver. We then utilized the well-established Drosophila Activity Monitor (DAM) System to record the locomotor activity of these flies in free-running conditions.

**Fig. 4 |:**
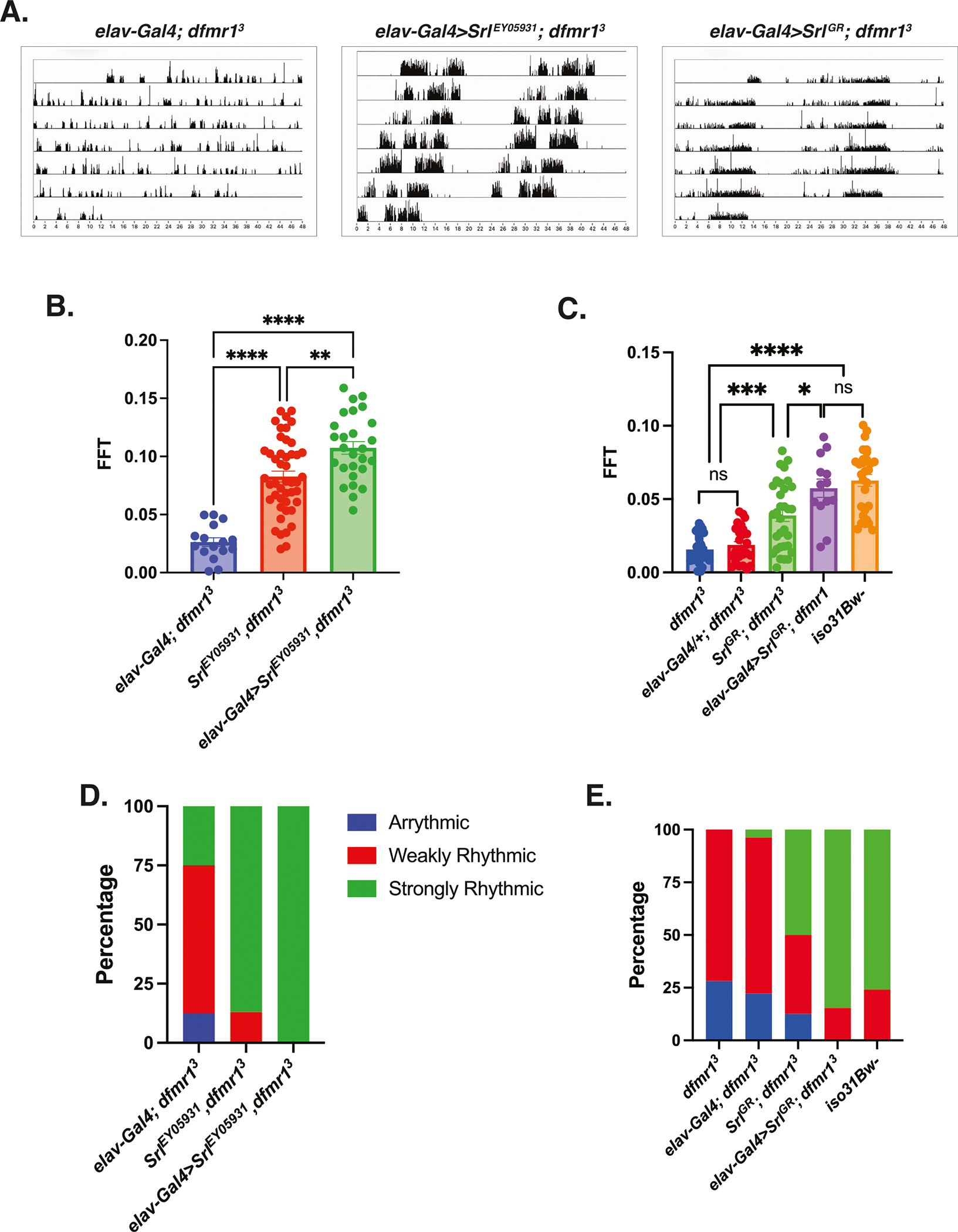
Pan-neuronal augmentation of Spargel rescues circadian rhythmicity in *dfmr1* mutant flies. **A–E** Circadian behavior was evaluated by comparison of actogram appearance, the average fast Fourier transform (FFT) values, and percentage of rhythmic flies to ascertain whether augmentation of *Spargel* expression improves circadian behavior in *dfmr1* mutant flies. Sample number (N) per genotype: (*elav-Gal4; dfmr1*) = 16–27; (*Srl*^*EY05931*^,dfmr1) = 46, (*elav*-*Gal4*>*Srl*^*EY05931*^,*dfmr1*) = 27, *dfmr1* = 32, (*Srl*^*GR*^*; dfmr1*) = 32, (*elav-Gal4*>*Srl*^*G*R^; *dfmr1*) = 13, *iso31Bw*- = 29. **A** Representative actograms from flies of the genotypes indicated. In contrast to the free-running rest:activity rhythms of *dfmr1* mutants that contain the *elav-Gal4* transgene alone, *dfmr1* mutants that contain the *Srl*^*EY05931*^ or *Srl*^*GR*^ transcript in conjunction with the *elav-Gal4* driver display consolidated, rhythmic behavior. **B, C** The average fast Fourier transform (FFT) was calculated for genetic combinations. One-way ANOVAs revealed a significant group effect for FFT values (*p* < 0.0001). Post hoc Tukey tests indicated that *dfmr1* mutant flies that expressed the (**B**) *Srl*^*EY05931*^ or (**C**) *Srl*^*GR*^ construct in conjunction with the *elav-Gal4* driver had significantly higher FFT values compared to *dfmr1* mutant flies that contained either transgene alone. Values represent mean ± SEM. *p ≤ 0.05, **p ≤ 0.01, ***p ≤ 0.001, ****p ≤ 0.0001. **D, E** The percentages of strongly rhythmic (FFT ≥ 0.04), weakly rhythmic (0.04 > FFT ≥ 0.01), and arrhythmic (FFT < 0.01) flies of each genotype are shown in green, red, and blue, respectively. The percentage of strongly rhythmic flies is increased in *dfmr1* mutants that contained the (**D**) *Srl*^*EY05931*^ or (**E**) *Srl*^*GR*^ construct in conjunction with the *elav-Gal4* driver compared to either construct alone.

Visual inspection of free-running rest:activity rhythms revealed that *dfmr1* mutant flies that contained the *elav-Gal4* driver in conjunction with the *Srl*^*EY05931*^ transgene had more rhythmic patterns of locomotor activity compared to *dfmr1* mutants that contained the *elav-Gal4* driver alone (Fig. 4A). To quantify circadian rhythmicity, we calculated fast Fourier transform (FFT) values for flies of each genotype. Conventionally, a fly with an FFT value that is above 0.04 is considered strongly rhythmic. In contrast, flies with FFT values between 0.01 and 0.04 are considered weakly rhythmic and those with FFT values below 0.01 are designated as arrhythmic. Congruent with their actogram appearance, on average, flies that pan-neuronally expressed the *Srl*^*EY05931*^ transgene had higher FFT values than their *dfmr1* mutant counterparts that contained the elav-Gal4 driver or *Srl*^*EY05931*^ transgene alone (Fig. 4B). When we stratified the dataset into categories based on the percentage of rhythmic flies, we observed that in contrast to *dfmr1* mutants that expressed only the *elav-Gal4* driver or *Srl*^*EY05931*^ construct, all *dfmr1* mutant flies that contained both the *elav-Gal4* driver and the *Srl*^*EY05931*^ construct were strongly rhythmic (Fig. 4D).

To independently corroborate our findings, we obtained a *UAS-Spargel* genomic rescue fragment (*Srl*^*GR*^) to elevate *Srl* expression in *dfmr1* mutants. Similar to what we observed with the *Srl*^*EY05931*^ construct, pan-neuronal expression of *Srl*^*GR*^ in *dfmr1* mutants also resulted in more consolidated, rest: activity patterns than *dfmr1* mutant conspecifics that contained only the elav-Gal4 driver alone (Fig. 4A). Moreover, pan-neuronal expression of *Srl*^*GR*^ in *dfmr1* mutants increased the average FFT value and percentage of strongly rhythmic flies relative to *dfmr1* mutant flies that contained either transgene alone (Fig. 4C, E). The ability of two distinct genetic manipulations that augment *Srl* expression to ameliorate circadian behavior in *dfmr1* mutants strongly suggests that elevation of *Srl* expression is sufficient to restore circadian rhythmicity. Notably, we did observe a significant increase in circadian rhythmicity for *dfmr1* mutant flies that contained the *Srl*^*EY05931*^ or *Srl*^*GR*^ transgene alone compared to *dfmr1* mutants that contained only the *elav-Gal4* driver (Fig. 4). We believe that this is likely due to leaky expression of the UAS constructs whereby *Srl* expression is moderately increased, albeit not as highly as when the *elav-Gal4* driver is present. Therefore, the rescue of circadian rhythmicity by elevation of *Srl* expression in *dfmr1* mutants appears to occur in a dose-dependent manner.

Consistent with the known role of *Srl* as a modulator of mitochondrial biogenesis, in follow up TEM experiments, we observed that pan-neuronal expression of *Srl*^*GR*^ in *dfmr1* mutants substantially improved thoracic mitochondrial ultrastructure compared to *dfmr1* mutants that contained the *elav-Gal4* driver alone (Supplementary Fig. 6). The ability of pan-neuronal expression of *Srl*^*GR*^ to restore mitochondrial ultrastructure in the thorax of *dfmr1* mutants suggests that this rescue occurs in a cell-non-autonomous manner. Much like our circadian results, the rescue of mitochondrial ultrastructure by augmentation of *Srl* expression appears to be dose-dependent, as mitochondrial ultrastructure was modestly improved in *dfmr1* mutants that caried the *Srl*^*GR*^ construct alone. Given that proper mitochondrial ultrastructure is essential to support energy production, we believe that elevation of *Srl* expression likely ameliorates mitochondrial function in *dfmr1* mutants and thereby restores circadian behavior.

### Genetic manipulation of *Spargel* expression phenocopies the *dfmr1* mutant circadian defect

**Fig. 5 |:**
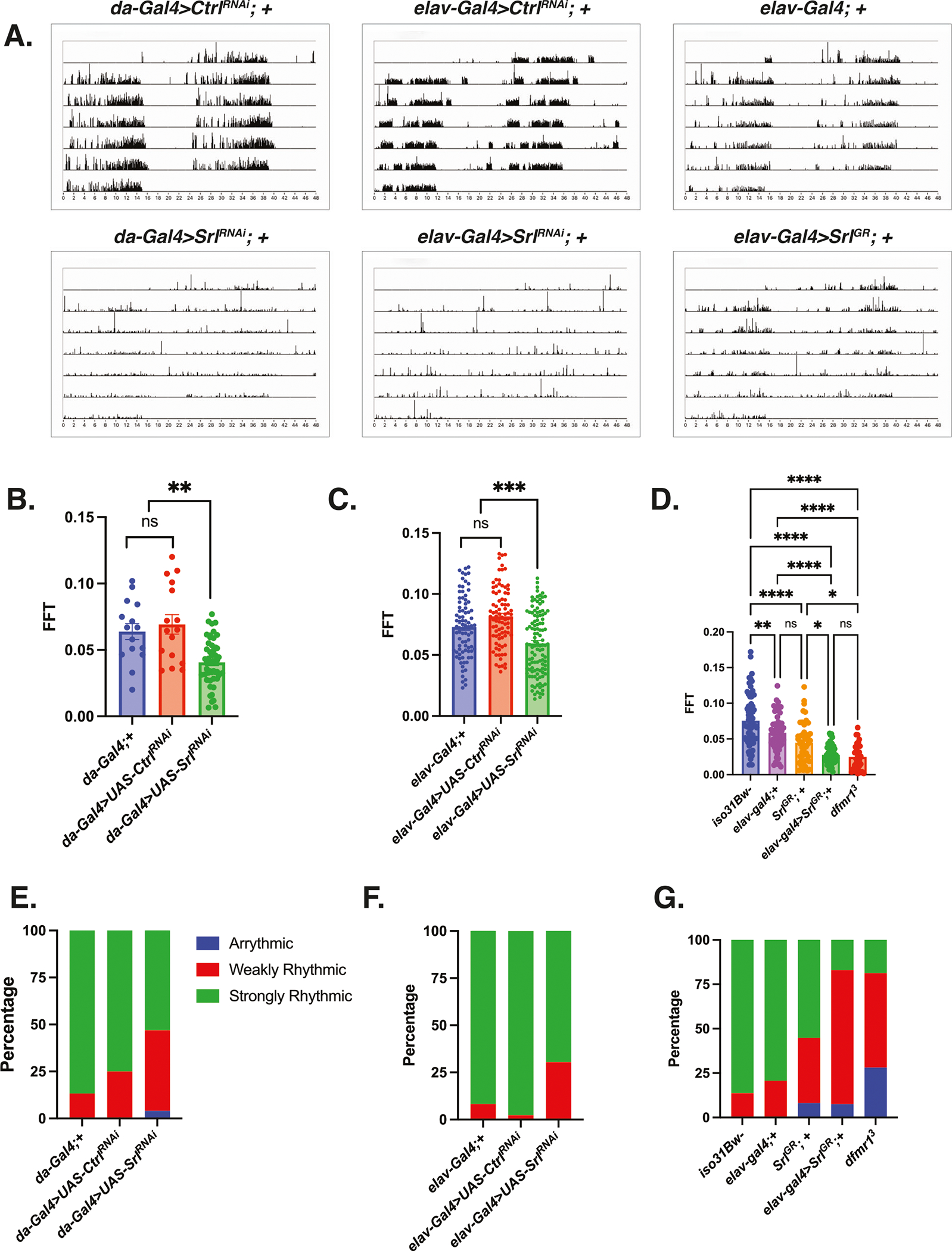
Modulation of *Spargel* expression in wild-type flies phenocopies the circadian defect observed in *dfmr1* mutant flies. **A–G** Circadian behavior was evaluated by comparison of actogram appearance, the average fast Fourier transform (FFT) values, and percentage of rhythmic flies for genetic combinations to ascertain whether overexpression or knockdown of *Spargel* impacts circadian behavior in wild type flies. Sample number (N) per genotype: (*da-Gal4*;+) = 15, (*da-Gal4 > UAS-Ctrl*^*RNAi*^) = 16, (*da-Gal4* > *UAS-Srl*^*RNAi*^) = 49, (*elav-Gal4*;+) = 58–84, (*elav-Gal4* > *UAS-Ctrl*^*RNAi*^) = 88, (*elav-Gal4* > *UAS-Srl*^*RNAi*^) = 115, (*Srl*^*GR*^; +) = 49, (*elav-Gal4*> *Srl*^*GR*^) = 53, *iso31Bw*- = 73, *dfmr1* = 32. **A** Representative actograms from flies of the genotypes indicated. While control flies in the top row (*da-Gal4* > U*AS-Ctrl*^*RNAi*^), (*elav-Gal4* > *UAS-Ctrl*^*RNAi*^), and (*elav-Gal4* > +) display consolidated, rhythmic rest: activity patterns, both Spargel loss-of-function (*da-Gal4* or *elav-Gal4* > *UAS-Srl*^*RNAi*^) and gain-of-function (*elav-Gal4>Srl*^*GR*^) disrupt rest:activity patterns. **B–D** The average fast Fourier transform (FFT) was calculated for genetic combinations. One-way ANOVAs revealed a significant group effect for FFT values (*p* < 0.0001). Post hoc Tukey tests indicated that wild-type flies that expressed a *UAS-Srl*^*RNAi*^ construct in conjunction with (**B**) *da-Gal4* or (**C**) *elav-Gal4* had significantly lower FFT values compared to wild-type flies that contained either Gal4 driver alone or a *UAS-Ctrl*^*RNAi*^ fragment. Similarly, (**D**) wild-type flies that pan-neuronally expressed a *Srl*^*GR*^ over-expression construct (*elav-Gal4*>*Srl*^*GR*^) had significantly lower FFT values than wild type flies that contained the *elav-Gal4* or *Srl*^*GR*^ transgene alone. There was no significant difference between the FFT values of the *elav-Gal4*> *Srl*^*GR*^ flies and those of *dfmr1* mutants. Values represent mean ± SEM. *p ≤ 0.05, **p ≤ 0.01, ***p ≤ 0.001, ****p ≤ 0.0001. **E–G** The percentages of strongly rhythmic (FFT ≥ 0.04), weakly rhythmic (0.04 > FFT ≥ 0.01), and arrhythmic (FFT < 0.01) flies of each genotype are shown in green, red, and blue, respectively. The percentage of weakly rhythmic and arrhythmic flies is increased in genetic combinations that result in (**E, F**) *Spargel loss*-of-function (*da-Gal4 or elav-Gal4* > *UAS-Srl*^*RNAi*^) and (**G**) gain-of-function (*elav-Gal4> Srl*^*GR*^) compared to flies that contain each transgene alone.

As further confirmation that *Srl* is indeed a major contributor to FXS pathophysiology, particularly with respect to circadian behavior, we next tested whether *Srl* loss-of-function in a wild-type genetic background phenocopies *dfmr1* loss-of-function. For these experiments, we used the *daughterless-Gal4* (*da-Gal4*) driver to ubiquitously express a *UAS-Srl*^*RNAi*^ transgene in an otherwise wild-type genetic background. When we assayed circadian behavior, we observed that in contrast to the empty vector control (*UAS-Ctrl*^*RNAi*^), ubiquitous knockdown of *Srl* expression in wild-type flies significantly disrupted free-running locomotor activity rhythms, diminished FFT values, and increased the percentage of flies that were arrhythmic or weakly rhythmic (Fig. 5A, B, E). Considering that pan-neuronal elevation of *Srl* expression was sufficient to rescue the circadian behavioral defect in *dfmr1* mutants, we hypothesized that *Srl* knockdown exclusively in the CNS would also mimic the *dfmr1* mutant phenotype. As expected, we observed that pan-neuronal knockdown of *Srl* expression in the wild-type genetic background recapitulated the circadian phenotype that we observed with the *da-Gal4* driver (Fig. 5A, C, F).

The ability of *Srl* loss-of-function to phenocopy the circadian defect that we observe in the *dfmr1* mutants prompted us to conduct the inverse experiment to query whether pan-neuronal over-expression of *Srl* also impacts circadian behavior. Indeed, we observed that targeted overexpression of the *Srl*^*G*R^ in the CNS alone results in significantly altered rest:activity rhythms, diminished FFT values, and an increased percentage of weakly rhythmic and arrhythmic flies (Fig. 5A, D, G). While it is counterintuitive that both loss and gain of function manipulations of *Srl* expression have the same impact on circadian behavior, we postulate that the maintenance of circadian rhythmicity is particularly sensitive to *Srl* dosage. In this way, both too much and too little *Srl* expression are detrimental to circadian behavior. Taking the loss- and gain- of function experiments together, it is clear that we have identified a novel role for *Srl* as a modulator of circadian behavior. This finding is particularly exciting because it positions *Srl* at the intersection of metabolism, mitochondrial function, and behavior.

## Discussion

While the causal gene was first cloned and identified nearly three decades ago, there is still much to be learned about the molecular underpinnings of FXS. Our incomplete understanding of the precise mechanisms that underlie FXS pathogenesis has precluded the identification of effective therapeutic approaches to ameliorate the quality of life of affected individuals. Despite the advancement of several compounds to clinical trials, findings at the bench have had mixed success at the bedside. Rather, it has become evident that the FXS field would benefit from the optimization of preclinical clinical strategies to identify therapeutic candidates and paradigms to predict their clinical efficacy.

One promising route to identify treatments for FXS has been the identification of conserved signaling pathway defects, such as IS, that can be targeted therapeutically^26^. Our studies in the *Drosophila* model of FXS pioneered the contribution of dysregulated IS to FXS pathogenesis^32^. In subsequent follow-up studies, we encountered a metabolic paradox whereby *dfmr1* mutant flies have decreased energy stores and are more sensitive to starvation despite elevated IS in the brain and hyperphagia^42^. Pursuit of an explanation for our discordant metabolic findings lead to our discovery that mitochondria in the *dfmr1* mutants have ultrastructural and functional defects^42^. Studies by other groups further support the notion that mitochondrial dysfunction is a robust, evolutionarily conserved component of FXS pathophysiology^49,51,58^.

The studies described herein integrate biochemical and physiologic methodologies with behavioral testing to provide a more comprehensive understanding of the contribution of mitochondrial dysfunction to FXS pathogenesis. We demonstrate that mitochondrial volume is diminished in the IPCs of *dfmr1* mutants and that ATP levels are decreased in *dfmr1* mutants compared to wild-type conspecifics. Strikingly, we report that genetic reduction of IS is sufficient to correct morphologic defects in the mitochondria of *dfmr1* mutants as well as augment the NAD^+^/NADH ratio and ATP levels. As such, our findings indicate that the defect in IS that modulates behavior and cognition in preclinical models of FXS is mechanistically linked to the observed mitochondrial defects. Further, we present evidence that dysregulated IS down-regulates the expression of the mitochondrial master regulator PGC-1α/Srl in the heads of *dfmr1* mutants. This result led to the exciting discovery that pan-neuronal augmentation of *Srl* expression is sufficient to mitigate circadian behavior in *dfmr1* mutants. Moreover, genetic manipulation of *Srl* expression in wild-type flies is sufficient to disrupt circadian behavior. Thus, in addition to implicating *Srl* in FXS pathogenesis, our findings reveal novel role of *Srl* in the regulation of circadian behavior. Future studies will be necessary to elucidate the precise mechanisms by which dysregulated IS impinges on PGC-1α expression in the brain. Additionally, it would be interesting to determine the extent to which mitochondrial phenotypes are present in female *dfmr1* mutants.

Notably, diminished PGC-1α expression has been reported in other syndromic forms of intellectual disability (ID) and autism, including Down Syndrome and Rett Syndrome^43,59^. The convergence of multiple distinct genetic forms of ID and autism on decreased PGC-1α expression suggests shared mechanistic underpinnings. Therefore, in a broader sense, further exploration of the precise mechanisms by which PGC-1α expression is compromised in ID and autism will expand our understanding of the contribution of reduced PGC-1α expression to behavioral pathology. Such studies have the potential to uncover novel treatments for FXS and other syndromic forms of ID and autism.

## Methods

### Fly genetics and husbandry

Fly strains that contain the *dfmr1*^*3*^ allele are described in Dockendorff et al. ^22^. Fly strains that contain the *dilp2* mutation were obtained from the Bloomington Stock Center (stock number 30881). The *elav-Gal4* and *daughterless-Gal4* drivers were derived from the Bloomington Stock Center (stock numbers 8765 and 95282). Flies that contain the *UAS-mitoGFP* construct were obtained from the Bloomington Stock Center (stock number 8442). The *UAS-Srl*^*RNAi*^ and *UAS-Ctrl*^*RNAi*^ empty vector control lines were obtained from the Vienna Drosophila Resource Center (stock numbers 330271 and 60200). Stocks that contain the *Srl*^*EY05931*^ allele were Bloomington Stock Center (stock number 20009). Stocks that contained the *UAS-Spargel*^*GR*^ were obtained from Hugo Stocker. All fly strains were outcrossed to *w1118* (*iso31Bw*-) flies as described in Monyak et al.^32^. Flies were cultured on a standard cornmeal-molasses medium and maintained in the presence of stringent 12 h light: 12 h dark (LD) cycles at 25 °C.

### Mitochondrial morphology in IPCs

We expressed the *UAS-mitoGFP* reagent described in^47^ under the control of the *dilp2-Gal4* driver to directly visualize mitochondria in the IPCs. Brains were dissected from 4 to 8 days old male flies in 1x PBS and fixed with 4% paraformaldehyde (PFA) in PBS for 20 min at RT. Brains were then washed 3 times for 10 min in PBS-T (PBS + 0.2% Triton-X 100) and mounted on slides in glycerol +2% N-Propyl gallate. Images were acquired with a Perkin Elmer UltraView Vox spinning disk confocal on a Nikon Eclipse Ti Microscope. Experiments were imaged on either a Hamamatsu EMCCD C9100–50 camera or a Hamamatsu CMOS ORCA-Fusion (C11440–20UP). The EMCCD camera was used with Volocity Software [Quorom Technologies/PerkinElmer] and the CMOS camera was used with VisiView (Visitron). Z-stacks encompassing mitoGFP signal were collected at 200-nm step-size. Images were analyzed using ImageJ (NIH). For each brain, a 50 μm long region encompassing the IPCs was cropped for analysis. Mitochondria were manually measured to determine their number and size. Mitochondrial signal was converted to a binary mask using the Pixel classification module of Ilastik, a machine-learning based image segmentation program^60^. We then used the 3D objects counter function in ImageJ to identify mitochondria and measure the volume per mitochondrion and total mitochondrial volume.

### NAD^+^/NADH quantification

The concentrations of nicotinamide nucleotides were measured using the NAD^+^/NADH Quantification Colorimetric Kit (Abcam, Waltham, MA) as described in^61^. Briefly, adult male flies aged 5 to 7 days were collected on dry ice. Fly heads were removed prior to homogenization and the decapitated fly bodies were pooled in groups of 10. The samples were homogenized in 400 μL of the NADH/NAD Extraction Buffer supplied in the kit and the homogenate was centrifuged at 18,407 × g for 5 min at 25 °C to remove debris. The cycling reaction was carried out as per the manufacturer’s instructions for 2 h and the nicotinamide nucleotide concentrations were determined in duplicate. The protein concentration of each sample was measured with the Pierce^®^ BCA Protein Assay Kit (Thermo Scientific, Rockford, IL). The concentration of nicotinamide nucleotide contained in each sample was then normalized to its respective protein content.

### ATP measurement

ATP levels were measured using the ATP Determination Kit (Molecular Probes, Eugene, Oregon) as described in^62^. Adult male flies aged 5 to 7 days were collected on dry ice. Fly heads were removed prior to homogenization and the decapitated fly bodies were pooled in groups of five. The samples were homogenized in 100 μL of ATP homogenization buffer [6 M guanidine HCL, 100 mM Tris (pH7.8), 4 mM EDTA]. An aliquot was boiled at 100 °C for 5 min and centrifuged for 3 min at maximum speed at 4 °C.10 μL of the supernatant was transferred to a 1.5 mL microfuge tube and diluted 1:10 with 90 μL dilution buffer [25 mM Tris (pH 7.8, 100 μM) EDTA]. Subsequently, 10 μL of the diluted supernatant was transferred to another 1.5 ml tube that contained 740 μL of dilution buffer such that the final dilution was 1:750. The diluted homogenate was centrifuged at 20,000 × g for 3 min. To prepare a series of low-concentration ATP standards, the 5 mM ATP stock solution provided with the kit was diluted with ddH_2_O to reach final concentrations of 0, 0.01, 0.05, 0.1, 0.5, and 1 μM. Next, 10 μL of each standard or sample was transferred in duplicate to a white, opaque 96 well plate. The reaction was started by adding 100 μL of the reaction mixture with a multichannel pipette. Luminescence was measured three times sequentially using a plate reader and the values were averaged. The protein concentration of each sample was measured with the Pierce^®^ BCA Protein Assay Kit (Thermo Scientific, Rockford, IL). The concentration of ATP contained in each sample was then normalized to its respective protein content.

### Transmission electron microscopy

Tissues for electron microscopic examination were prepared as described in^42^. Thoraces were fixed with 2.5% glutaraldehyde, 2.0% paraformaldehyde in 0.1 M sodium cacodylate buffer, pH7.4, overnight at 4 °C. After subsequent buffer washes, the samples were post-fixed in 2.0% osmium tetroxide for 1 h at room temperature, and then washed again in buffer followed by dH_2_O. After dehydration through a graded ethanol series, the tissue was infiltrated and embedded in EMbed-812 (Electron Microscopy Sciences, Fort Washington, PA). Thin sections were stained with lead citrate and examined with a JEOL 1010 electron microscope fitted with a Hamamatsu digital camera and AMT Advantage image capture software.

### Western analysis

Adult male flies aged 5–7 days were snap frozen and heads were separated into groups of 10 on dry ice. Protein extracts were prepared from the heads using extraction buffer 20 mM Hepes (pH 7.5), 100 mM KCl, 5% glycerol, 100 μM NA3VO4, 10 mM EDTA, 0.1% Triton X, 1 mM DTT, and (Phosphatase/protease inhibitors) 4X LDS (Invitrogen) and 10X Reducing Agent (Invitrogen) were added before samples were incubated at 70 °C for 10 min to denature and reduce. Samples were separated on a 4–12% Bis-Tris gel (Invitrogen) and transferred to a PVDF membrane (Immobilon-P, Millipore, St. Louis, MO). Enhanced chemiluminescence (SuperSignal West Pico; Thermo Scientific) was used for antibody detection. The following primary antibodies were used: anti-PGC-1α 1:1000 (Millipore, St. Louis, MO), anti-β-tubulin E7 1:20,000 (Developmental Studies Hybridoma Bank, Iowa City, IA). The relative intensity of PGC-1α to β-tubulin was determined using ImageJ (NIH).

### Circadian behavior

Circadian analysis was performed as described in Dockendorff et al., 2002. Male flies were collected 0–3 days post eclosion and entrained to a stringent 12 h light: 12 h dark cycle for three days at 25 °C. Flies were then placed in individual tubes containing 5% sucrose, 2% agar, and loaded into monitors (Trikinetics, DAM2 system, Waltham, MA) that were placed in an incubator in constant darkness at 25 °C. The activity of these flies, as indicated by beam breaks, was measured from days 2 to 6. Data were collected in 5 min bins and analyzed with ClockLab software (Actimetrics, Wilmette, IL). Rhythmicity was determined by a FFT analysis.

### Statistics

The Prism software package (GraphPad Software, v9.5.1) was used to generate graphs and perform statistical analyses. Unpaired t-tests were used to evaluate pairwise comparisons. Multiple comparisons were investigated using one-way analysis of variance (ANOVA) with post hoc Tukey tests. Multiple comparisons were investigated for datasets with variable standard deviations using Brown-Forsythe and Welch ANOVA with Dunnett’s T3 multiple comparisons test.

## Supplementary Material

Supplementary Figures

## Data Availability

The authors declare that the data supporting the findings of this study are available within this manuscript and the accompanying supplementary information files.
