# Supplementary material for: PGC-1α integrates insulin signaling with mitochondrial physiology and behavior in a Drosophila model of Fragile X Syndrome
Source: NPJ Metab Health Dis. Author manuscript; Available in PMC 2024 May 13. (PMC11090494; doi:10.1038/s44324-024-00004-7)
Supplement: Supplementary Figures [file NIHMS1986116-supplement-Supplementary_Figures.pdf]

## Supplementary Figure 1

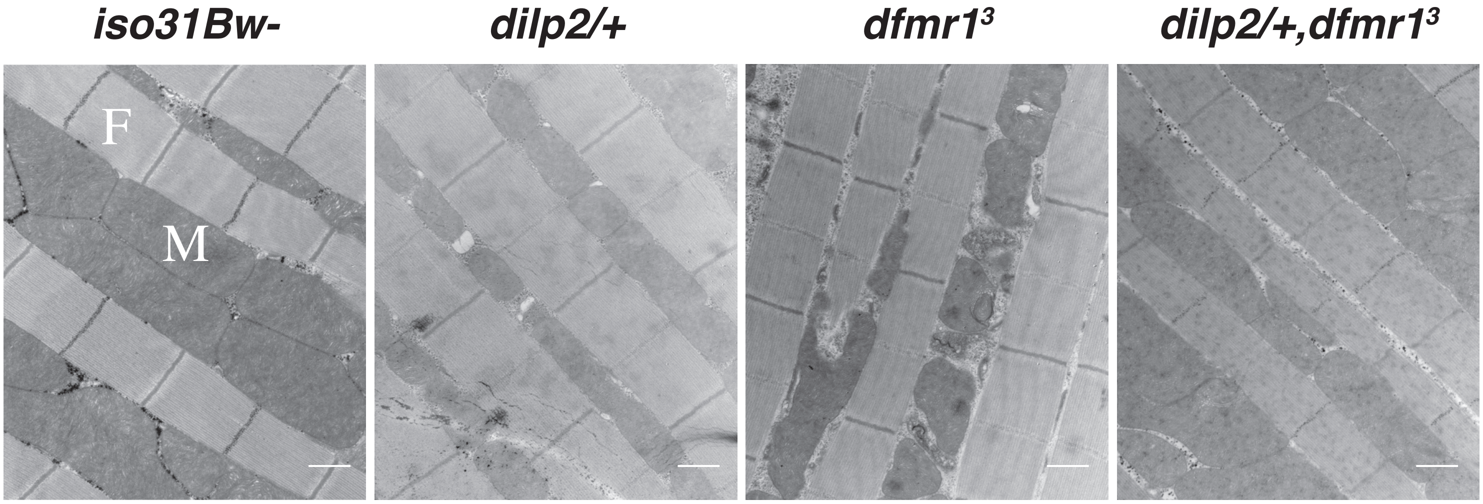

**Supplementary Figure 1.** Longitudinal sections of indirect flight muscle were prepared from isolated thoraces for transmission electron microscopy experiments: Electron micrographs of *Drosophila* flight muscle at 20,000x magnification. The genotype of each fly is denoted above its corresponding panel, from left to right: *iso31Bw-* control, heterozygous *dilp2*/+ single mutant, homozygous *dfmr1* single mutant, and *dilp2/+*, *dfmr1* double mutant. Scale bar indicates 800 nm. Mitochondria, M, are aligned between rows of myofibrils, F. The mitochondria of the *dfmr1* mutants appear smaller and contain ultrastructural defects, particularly dispersions of cristae compared to *iso31Bw-* controls. In contrast, the mitochondria of the *dilp2/+*, *dfmr1* double mutants appear larger and have ultrastructure that more closely resembles that of *iso31Bw-* conspecifics than that of *dfmr1* single mutants.

## Supplementary Figure 2

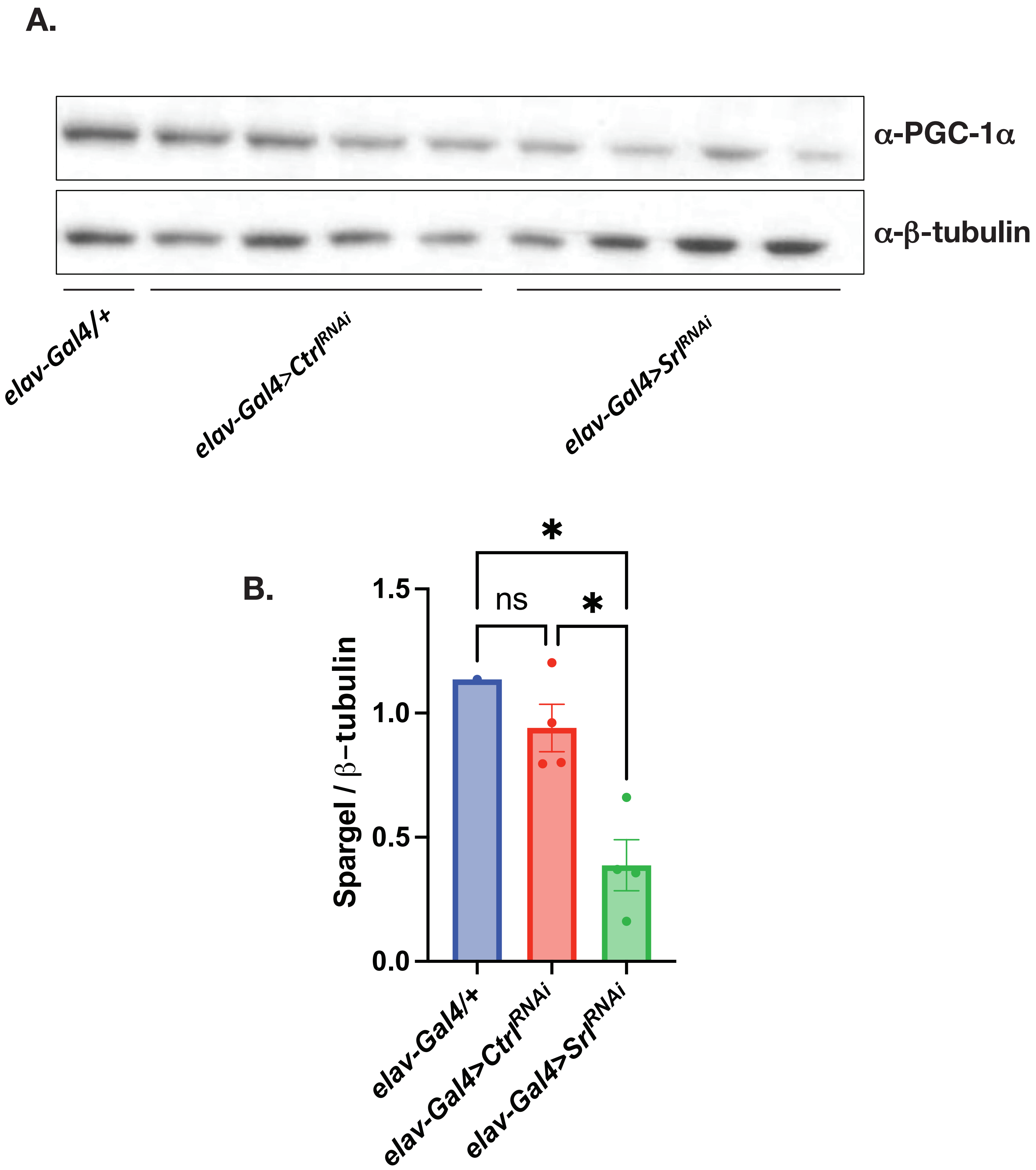

**Supplementary Figure 2. (A)** Western analysis of Spargel expression in head extracts from wild type flies that either contained the *elav-Gal4* driver alone (*elav-Gal4*/+); the *elav-Gal4* transgene and a *UAS-Ctrl*$^{RNAi}$ construct (*elav-Gal4> UAS-Ctrl*$^{RNAi}$); or the *elav-Gal4* transgene and a *UAS-Srl*$^{RNAi}$ construct (*elav-Gal4> UAS-Srl*$^{RNAi}$). An antibody to PGC-1α was used to detect Spargel expression (top). β-Tubulin was used as a loading control (bottom). **(B)** Quantification of the intensity of Spargel relative to β-Tubulin. A one-way ANOVA revealed a significant group effect for Spargel expression relative to β-Tubulin ($p=0.0116$). Post hoc Tukey tests indicated that the *elav-Gal4>UAS-Srl*$^{RNAi}$ flies had significantly lower Spargel levels than *elav-Gal4*/+ and *elav-Gal4> UAS-Ctrl*$^{RNAi}$ controls. Sample number (N) per genotype: (*elav-Gal4*/+)= 1, (*elav-Gal4> UAS-Ctrl*$^{RNAi}$)= 4, and (*elav-Gal4> UAS-Srl*$^{RNAi}$)= 4. Each sample contained 10 fly heads. Values represent mean ± SEM. *$p \leq 0.05$. As predicted, we observed a strong band of the correct size whose expression relative to β-Tubulin decreased when we pan-neuronally expressed a *UAS-Srl*$^{RNAi}$ construct (*elav-Gal4>UAS-Srl*$^{RNAi}$) compared to flies that pan-neuronally expressed the VDRC library control RNAi construct (*elav-Gal4> UAS-Ctrl*$^{RNAi}$) or *elav-Gal4* driver alone.

## Supplementary Figure 3

**A.**

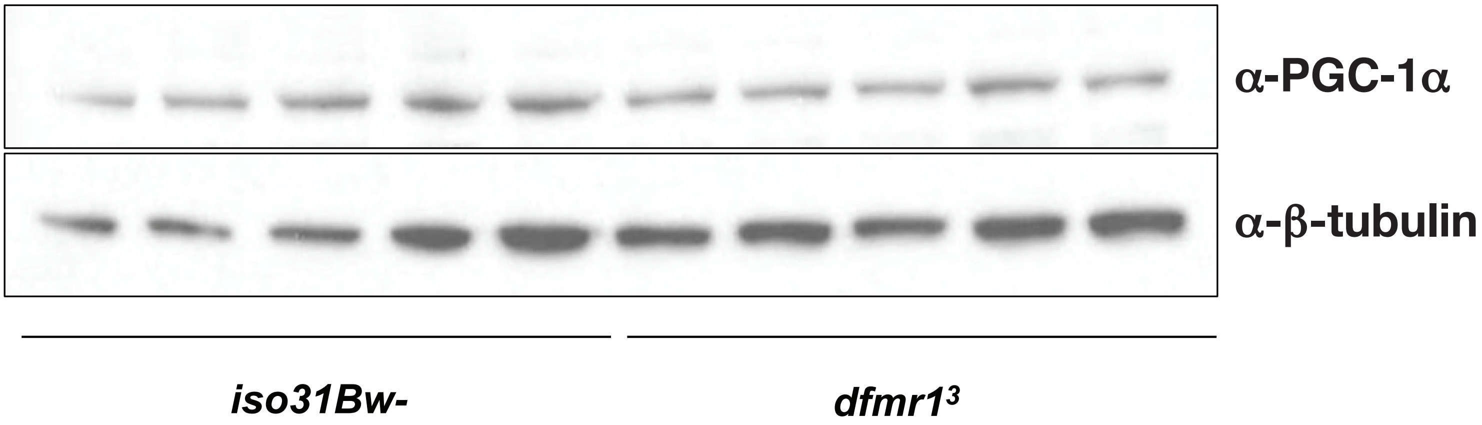


**B.**

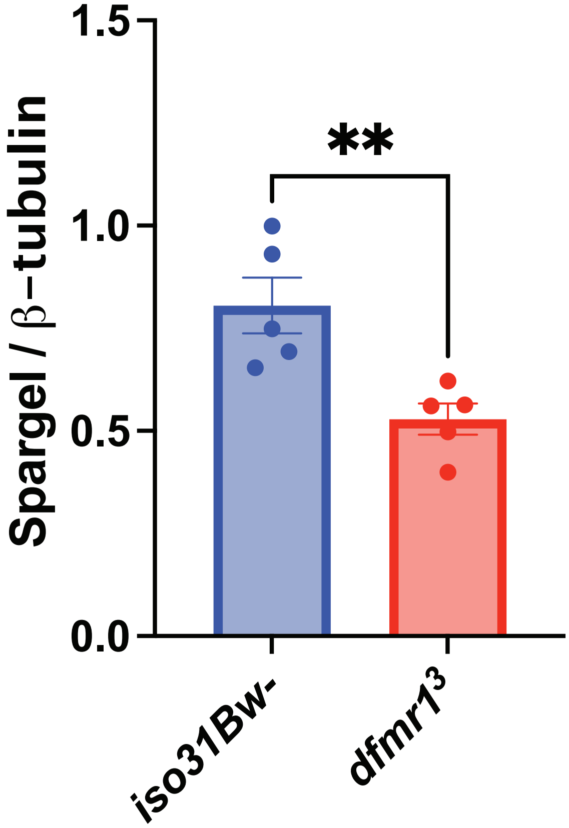

**Supplementary Figure 3**

**(A)** Western analysis of Spargel expression in extracts from *iso31Bw-* and *dfmr1* mutant fly heads. An antibody to PGC-1α was used to detect Spargel expression (top). β-Tubulin was used as a loading control (bottom). **(B)** Quantification of the intensity of Spargel relative to β-Tubulin. An unpaired t-test revealed that Spargel levels are diminished in *dfmr1* mutant heads compared to *iso31Bw-* controls ($p=0.0074$). Sample number (N) per genotype =5. Each sample contained 10 fly heads. Values represent mean ± SEM. $^{**}p \leq 0.01$

## Supplementary Figure 4

**A.**

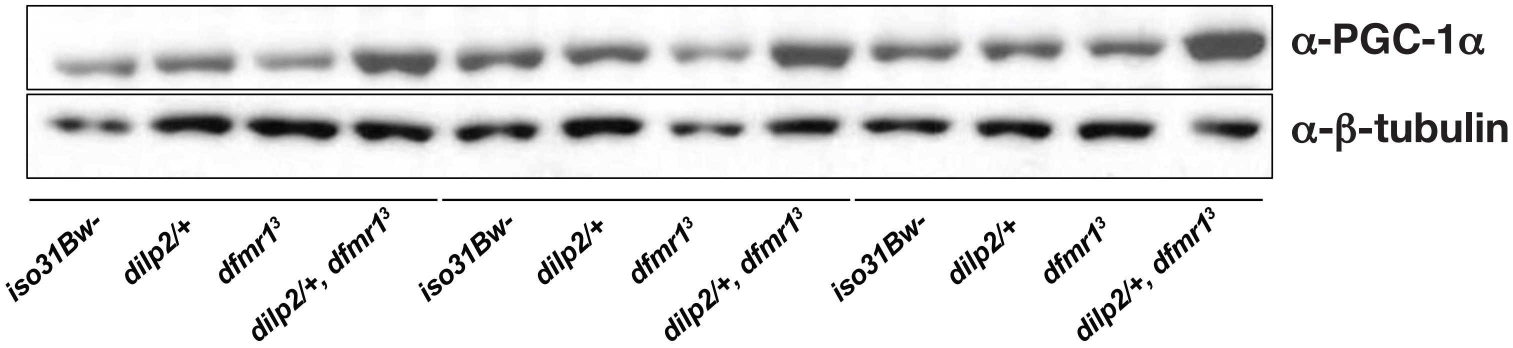


**B.**

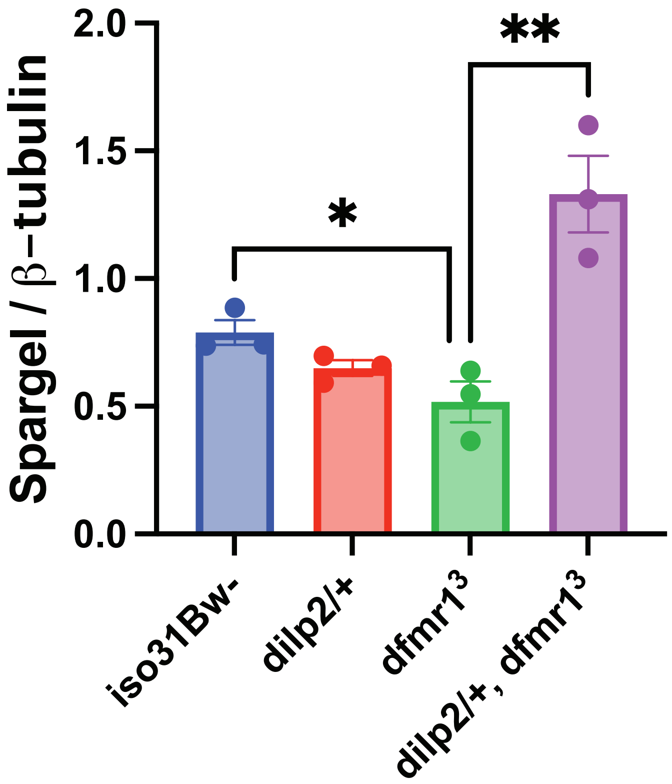

**Supplementary Figure 4**

**(A)** Western analysis of Spargel expression in extracts from *iso31Bw-* wild type, *dilp2/+* heterozygous mutant, *dfmr1* homozygous mutant, and *dilp2/+, dfmr1* double mutant fly heads.

**(B)** An unpaired t-test indicated that flies *dilp2/+*, *dfmr1* double mutant flies have higher Spargel expression than *dfmr1* single mutants (p=0.0088). Sample number (N) per genotype =3. Each sample contained 10 fly heads. Values represent mean ± SEM. **p≤0.01

## Supplementary Figure 5

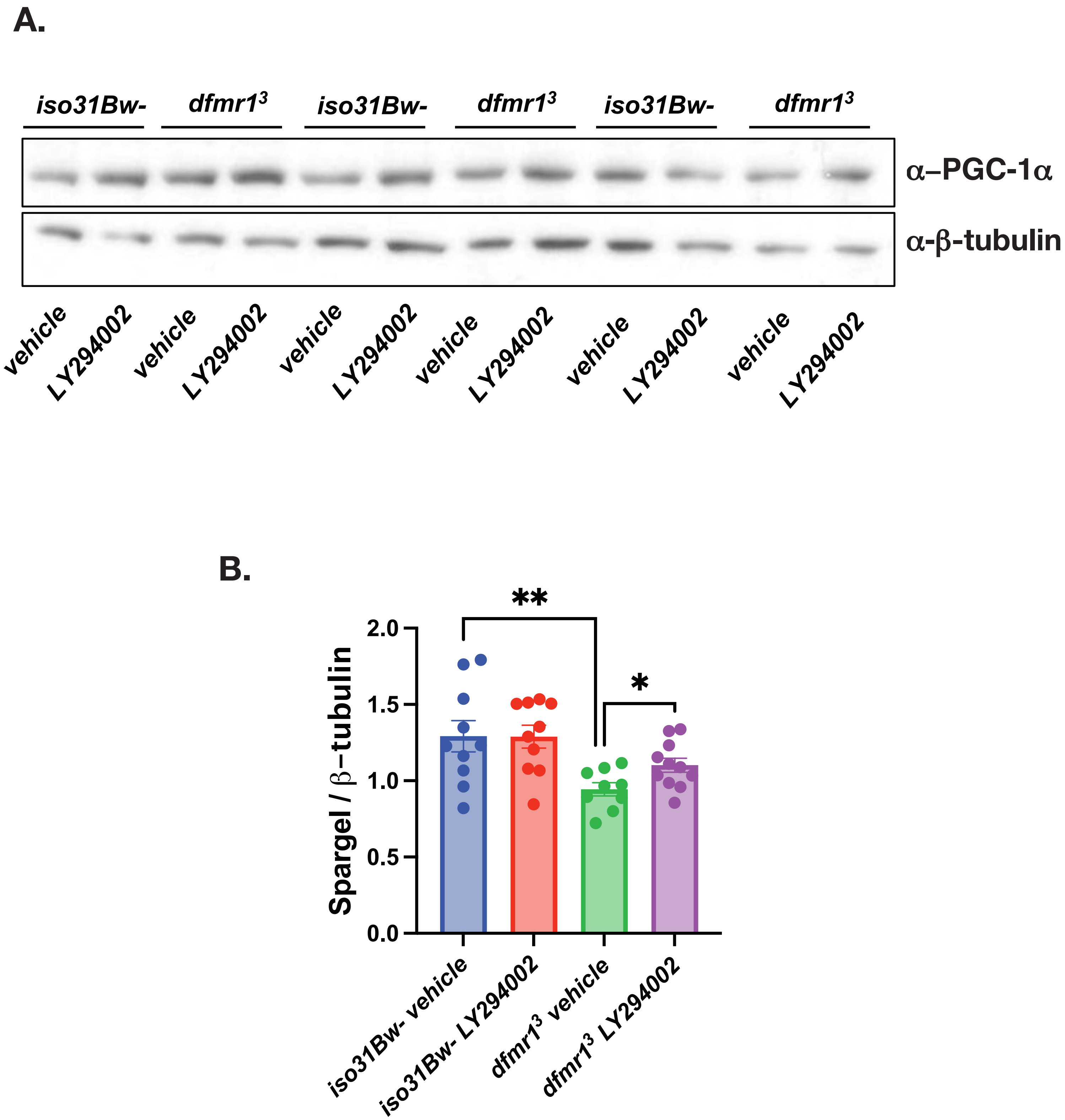

**Supplementary Figure 5**

**(A)** Representative western analysis of Spargel expression in extracts from *iso31Bw-* and *dfmr1* mutant fly heads that treated with either 5µM LY294002 or ethanol vehicle for 5 days post eclosion. An antibody to PGC-1α was used to detect Spargel expression (top). β-Tubulin was used as a loading control (bottom). **(B)** Quantification of the intensity of Spargel relative to β-Tubulin. Results from three independent experiments were pooled together. An unpaired t-test indicated that *dfmr1* mutants treated with vehicle had lower Spargel expression than *iso31Bw-* controls treated with vehicle ($p=0.0079$). An unpaired t-test indicated that *dfmr1* mutant flies that were treated with LY294002 have higher Spargel expression than *dfmr1* mutants that were treated with vehicle ($p=0.0239$). Sample number (N) per condition: *iso31Bw-* (vehicle)=10, *iso31Bw-* (LY294002)=10, *dfmr1* (vehicle)=9, *dfmr1* (LY294002)=11. Each sample contained 10 fly heads. Values represent mean ± SEM. *$p \leq 0.05$, **$p \leq 0.01$

## Supplementary Figure 6

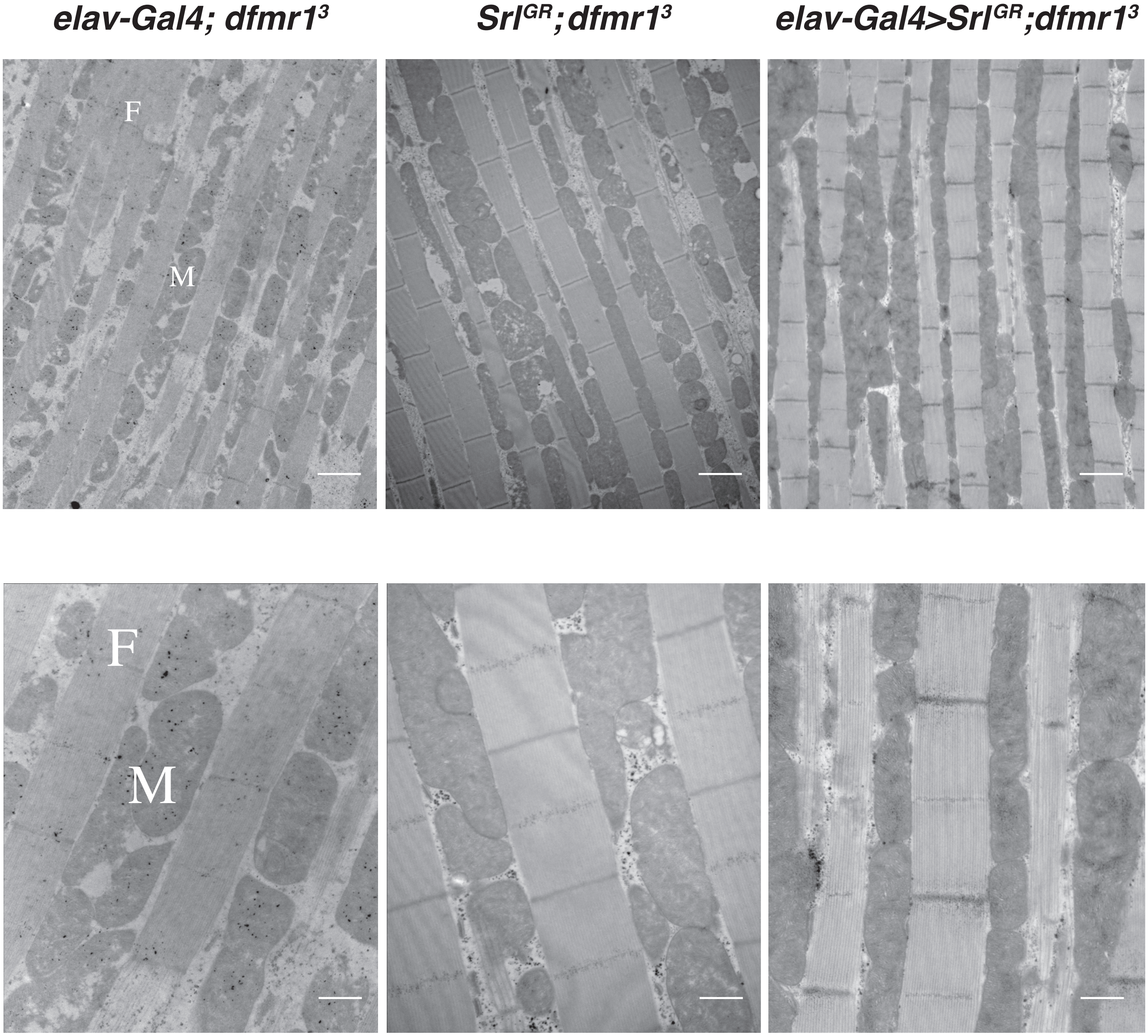

**Supplementary Figure 6**

Longitudinal sections of indirect flight muscle were prepared from isolated thoraces for transmission electron microscopy experiments: Electron micrographs of *Drosophila* flight muscle at 7,500x magnification (top) and 20,000x (bottom). The genotype of each fly is denoted above its corresponding panel, from left to right: (*elav-Gal4; dfmr1*$^{3}$), (*Srl*$^{GR}$*; dfmr1*$^{3}$), and (*elav-Gal4> Srl*$^{GR}$*; dfmr1*$^{3}$). Scale bar indicates 2 microns for 7,000x magnification panels and 800 nm for 20,000x magnification panels. Mitochondria, M, are aligned between rows of myofibrils, F. The mitochondria of the *dfmr1* mutants that contain the *elav-Gal4* driver alone appear to be small and sparsely distributed along the adjacent myofibrils. These mitochondria also contain ultrastructural defects, particularly dispersions of cristae. In contrast, the mitochondria of the *dfmr1* mutants that contain the *Srl*$^{GR}$ construct in conjunction with the *elav-Gal4* driver appear to be larger and fill the majority of the space between myofibrils. These mitochondria also contain more densely packed cristae that are free of dispersions. The mitochondria of *dfmr1* mutants that contain the *Srl*$^{GR}$ construct alone have an intermediate phenotype, whereby there appears to be some modest improvement in mitochondrial ultrastructure. However, these mitochondria are more variable in size with large gaps between the myofibrils and areas of dispersed cristae. Given that UAS elements are known to have leaky expression even in the absence of a Gal4 driver, we believe that the rescue of mitochondrial ultrastructure by the *Srl*$^{GR}$ construct alone likely results from low levels of leaky expression *Srl*$^{GR}$. The concordance between the dose-dependent rescue of circadian behavior and that of mitochondrial ultrastructure by *Srl*$^{GR}$ provides further evidence that these phenotypes are mechanistically linked.
